# Pedestrians’ Understanding of a Fully Autonomous Vehicle’s Intent to Stop: A Learning Effect Over Time

**DOI:** 10.3389/fpsyg.2020.585280

**Published:** 2020-12-03

**Authors:** Michal Hochman, Yisrael Parmet, Tal Oron-Gilad

**Affiliations:** Department of Industrial Engineering and Management, Ben-Gurion University of the Negev, Beersheba, Israel

**Keywords:** fully autonomous vehicle, external human-machine interfaces, presentation modality, road crossing, eye movements

## Abstract

This study explored pedestrians’ understanding of Fully Autonomous Vehicles (FAVs) intention to stop and what influences pedestrians’ decision to cross the road over time, i.e., learnability. Twenty participants saw fixed simulated urban road crossing scenes with a single FAV on the road as if they were pedestrians intending to cross. Scenes differed from one another in the FAV’s, distance from the crossing place, its physical size, and external Human-Machine Interfaces (e-HMI) message by background color (red/green), message type (status/advice), and presentation modality (text/symbol). Eye-tracking data and decision measurements were collected. Results revealed that pedestrians tend to look at the e-HMI before making their decision. However, they did not necessarily decide according to the e-HMIs’ color or message type. Moreover, when they complied with the e-HMI proposition, they tended to hesitate before making the decision. Overall, a learning effect over time was observed in all conditions regardless of e- HMI features and crossing context. Findings suggest that pedestrians’ decision making depends on a combination of the e-HMI implementation and the car distance. Moreover, since the learning curve exists in all conditions and has the same proportion, it is critical to design an interaction that would encourage higher probability of compatible decisions from the first phase. However, to extend all these findings, it is necessary to further examine dynamic situations.

## Introduction

Crossing the street in the Fully Autonomous vehicle (FAV) era will differ from road crossing today since, among other things, the crossing decision will not be influenced by informal pedestrian – driver human-human communication (like eye contact, facial expressions, gestures, or body movements) that is necessary to understand driver intention (Rasouli et al., 2018). Thus, in the FAV era, with the absence of a human driver, the main challenge would be to establish pedestrians’ understanding of FAV intentions so that they can make safe crossing decisions.

Simulation studies reported that an external human-machine interfaces (e-HMI) mounted on the vehicle enhances the interaction with pedestrians by reducing the uncertainty regarding FAV intent, improving pedestrians’ initial trust and understanding (Deb et al., 2018; Ackermann et al., 2019; Ackermans et al., 2020). It was claimed that pedestrians have high trust and confidence in the e-HMI, even before getting to know it, and they tend to comply with its instructions (Holländer and Butz, 2019). Moreover, even after a malfunction, trust and confidence recovered quickly (Holländer and Butz, 2019). Inconsistent with this claim, a Wizard of Oz (WoZ) study suggested that people prefer to decide for themselves when to cross, as they do today, based on the FAV’s distance and speed from their crossing point (Clamann et al., 2017). Another video-based study followed by questionnaires reported similar trends (Mahadevan et al., 2018).

Few studies dealt with the form of the visual e-HMI messages. One distinction is between advice messages that suggest to the pedestrian whether to cross the road or not (e.g., “please cross,” “walk,” “stop”) and status messages that display the FAV status, like “Driving,” “Stopping,” etc (Deb et al., 2018; Ackermann et al., 2019). Another distinction was between text and symbol messages (Deb et al., 2018; Ackermann et al., 2019). A study that looked at pedestrians’ comprehension of the e-HMI messages through questionnaires revealed that participants assessed advice messages as more comfortable than status messages, independent of text or symbol-based presentation (Ackermann et al., 2019). On the contrary, Deb et al. (2018) found that a textual “Braking” status message was preferred over textual advice “Walk” message.

Studies also varied in the way they measured pedestrians’ understanding. One way is to measure the time it took the pedestrian to decide whether to cross the road in a VR simulation (Clamann et al., 2017; Dey et al., 2018). Decision time was faster when e-HMI display included text or symbol compared to no e-HMI (Dey et al., 2018). Another way is through subjective questionnaires and ratings (Deb et al., 2018; Ackermann et al., 2019). A third way is through accuracy rate, that is, whether the pedestrian’s decision was in agreement with what was being displayed on the e-HMI [compatible responses, noted as the e-HMI proposition in Ackermann et al. (2019)]. This can also be measured through the error probability (i.e., the probability of incompatible responses), that is, decisions that were not in agreement with the e-HMI display.

When examining an e-HMI, it is essential to explore learnability. Learnability was found to significantly affect users adopting new technology and on user satisfaction from a product (Noel et al., 2005). Also, it was found that learnability directly influences safety when considering drivers (Noel et al., 2005). When investigating the learnability of a pedestrian’s interaction with a FAV, in a WoZ field experiment, researchers found a learning curve over time but in a rather limited form as the authors based the learning on rating questionnaires over time (Faas et al., 2020). Researchers investigated learnability with a single item: the participant agreement with the statement, “It is easy to learn that the light signal on the vehicle indicates yielding” (strongly disagree – strongly agree) while comparing steady, flashing, and sweeping light signals.

The current study aims to investigate factors that influence pedestrians’ understanding of a FAV’s intention by looking at their decisions and scanning patterns when aiming to cross the road, in fixed simulated scenes from the perspective of the pedestrian, in general, and over time. The factors examined are related to the characteristics of the e-HMI, color, message type (advice or status message) and modality (text or symbol), and the crossing context; FAV size and distance from the crossing place. Also, using eye-tracking to measure pedestrians’ visual attention distribution while deciding to cross is common in pedestrian behavior studies (e.g., Tapiro et al., 2016). Explicitly, it can indicate whether pedestrians looked at the e-HMI and for how long before the decision to cross or not. Field research investigated pedestrians’ gaze patterns, but only with a manual car that did not include e-HMI (Dey et al., 2019). Another research reported a negative correlation between pedestrians’ subjective understanding of the FAV intention and their gaze fixation duration (Liu et al., 2020). Furthermore, to our knowledge, the interaction between the crossing decision making (to cross or not cross) and pedestrians’ gaze behavior on the FAV’s e-HMI is yet to be investigated in general and learnability over time. Also, with regard to the measurement of response time and error probability.

The following hypotheses are suggested: h1- the e-HMI’s proposition would lead pedestrians to make more compatible decisions, particularly when it conflicts with the crossing conditions (e.g., short distance). This hypothesis is based on previous contradicting findings regarding what affects pedestrians to cross in the FAV world like distance (Clamann et al., 2017) or e-HMI proposition (Holländer and Butz, 2019). *h2*- Advice message would reduce error probability compared to status message (Ackermann et al., 2019). *h3*- is regarding learnability, we expect error probability and response time to reduce over time regardless of the crossing context and e- HMI display characteristics due to learnability.

## Materials and Methods

### Participants

Twenty students aged 21–34 (*M* = 26, SD = 3, 11 females) participated in the experiment. One participant’s data were excluded due to technical problems. As compensation, seven participants received course credit and 13 a payment of $10. All participants had normal contrast sensitivity and visual acuity of at least 6/6. Participants were free to withdraw from the study at any time.

### Apparatus

#### Experimental Environment

**FIGURE 1:**
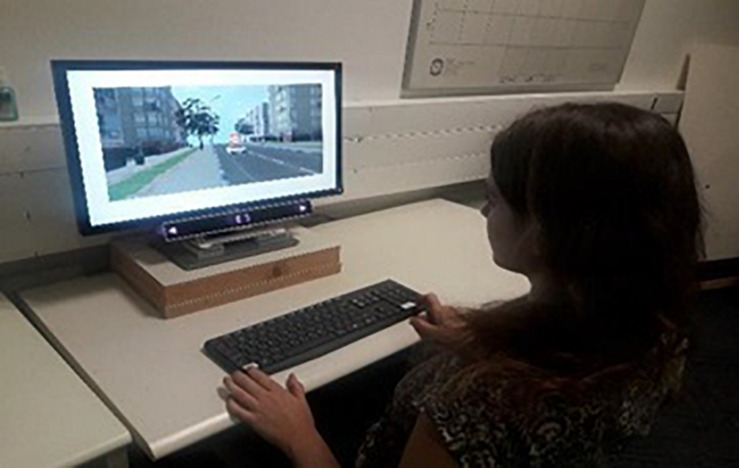
The experimental testbed, consisting of a 22″ screen, the Gaze point eye-tracking system and the keyboard to collect participants’ responses.

The study was conducted at the Eye Tracking laboratory using a desktop test station computer with a 22″ screen. Participants were situated approximately 70 cm from the screen. The Gaze point eye tracker was located below the screen (see Figure 1).

#### Fixed Scene Generation

**FIGURE 2:**
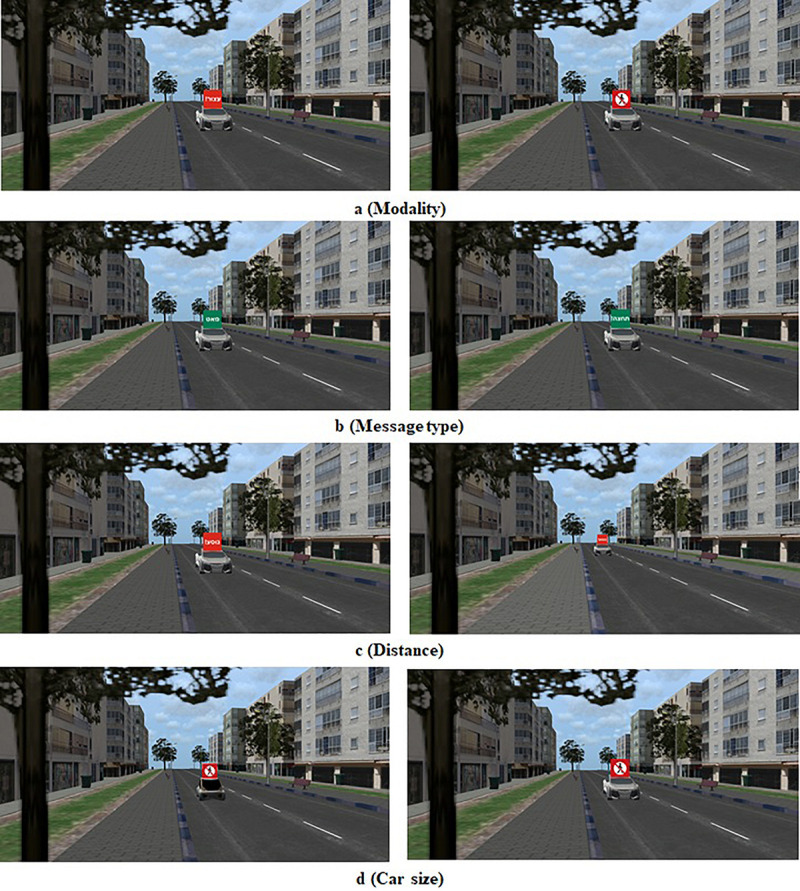
Sample crossing scenes, as seen from the perspective of the pedestrian. Each row **(a–d)** demonstrates an examined factor. **(a)** Modality: left- Text, right- Symbol. **(b)** Message type: left- Status, right- Advice. **(c)** Distance: left- close, right- far. **(d)** Car size: left – Small (Kancil),right- Large (Audi).

**FIGURE 3:**
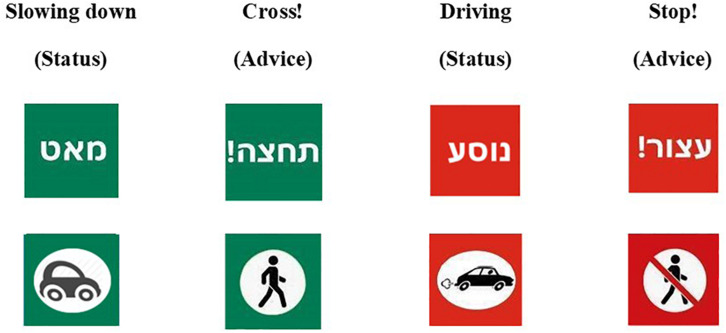
The e-HMI Messages; each column displays the message type (status or advice) in both written (in Hebrew) and symbolic messages. The background color is compatible with the message meaning according to the color convention.

One hundred and eight fixed scenes were generated using the VT-MAK VR tools^1^ with a typical local city’s 3D terrain model. The crossed road was a one direction two-lane urban road. To add realism to the scene, the city’s typography included buildings, light posts, vegetation, etc (Figure 2). The images were taken from the pedestrian’s perspective as if standing on the curb and looking to the left before crossing the street. Each image included a combination of a single FAV (small or big) on the closer lane, either far (20 m) or close (9 m) to the pedestrian’s crossing point in the simulation. The e-HMI size in the far distance was 0.9 cm × 0.9 cm and in the close distance 20 cm × 20 cm. The e-HMI was located on the roof of the car (this location was found to be very useful in previous research (Bazilinskyy et al., 2020). It included a sign that could convey either a written message (text) or a symbolized message (see Figures 2, 3). Also, the message content could be a status message (“Slowing” or “Driving”) or advice message (“Cross” or “Don’t Cross”). Also, the e-HMI background color was green or red. I previous research, it was found that color convention helped pedestrians understand the FAV intention; that is, a green e-HMI indicated it was s safe to cross, and the red implies that it was unsafe to cross (Rouchitsas and Alm, 2019; Bazilinskyy et al., 2020). Besides, baseline images without the e-HMI were created, with a variation of car size and crossing distance (for the content of the entire images, see Supplementary Appendix 1).

#### Eye-Tracking System

The Gaze point eye-tracking system was used to measure pupil diameter and gaze direction with an accuracy visual angle of 0.5–1 degree (Figure 1). The system uses an eye camera and an infra-red eye illuminator to sample a close image pupil at a sample rate of 60 Hz.

### Road Crossing Task

**FIGURE 4:**
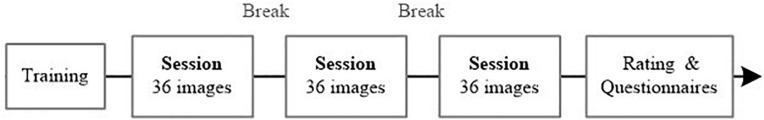
The experimental flow.

Each participant took part in three consecutive sessions (Figure 4). In each session, participants were asked to observe 36 consecutive crossing scenes and decide for each one, as quickly as possible, if it was safe to cross the road or not. The decision was made by selecting the “Safe to cross” or “Not safe to cross” designated keyboard buttons.

### Dependent Variables

#### Estimated Error Probability

An error was defined as the incompatibility of the participant’s selection (whether to cross or not) with the sign meaning (as *a priori* defined). If the selection had not the same value (safe/unsafe), it was counted as an error. In the model, we predicted the estimated error probability. Within the images that had no e-HMI, the incompatible response was unknown, and therefore, the error probability was undefined.

#### Response Time

Time from the moment the image was displayed until the participant pressed a decision button.

#### Eye-Tracking Measures

**FIGURE 5:**
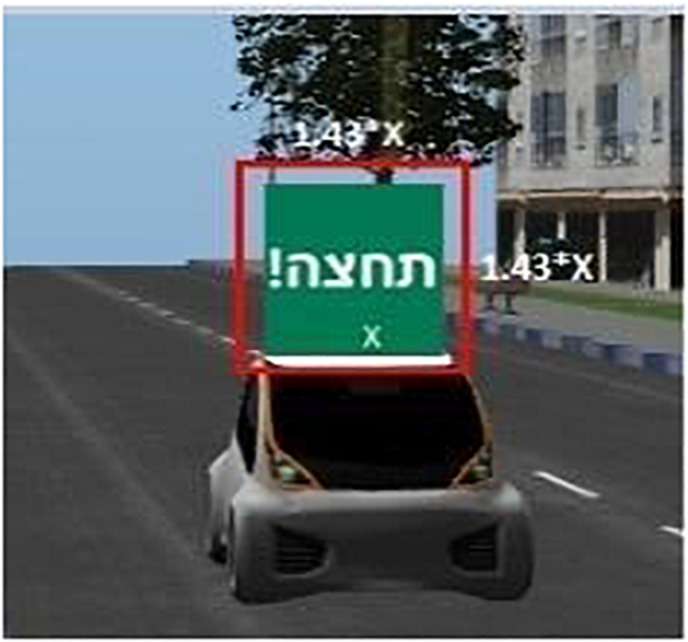
Defining the AOI around the e-HMI.

Total fixation duration and the total number of fixations on the e-HMI. A fixation was defined as a period of at least 100 ms that the eyes remain relatively still. The gaze data is based on the position variance technique (Jacob, 1995), that is, a sequence of gaze data estimates spatially located within a local region are determined to belong to the current fixation, while subsequent data outside of this local region is identified as the beginning of a new fixation. The fixations counted were only the ones within the area of interest (AOI), which was defined as the e-HMI sign (Figure 5).

#### Learnability

Learnability was defended as the improvement in performance over time, from the trial to trial, that is, the reduction in the error probability, response time, and the number of fixations.

#### Subjective Measurements

A written explanation of the sign meaning and rating its comprehension level (on 10-point rating scale), followed by an open interview on how each participant made their crossing decisions.

### Experimental Design

A within-subject design. The following independent variables were defined: e-HMI (included/none), message type (status message/advise message), modality (text/symbol), car size (big/small), color (red/green) and car distance (close/far), altogether a 2^∧^6 factorial design.

### Procedure

Participants were invited individually to the lab for approximately 30 min. Following instructions and signing a consent form, they performed visual acuity and contrast sensitivity tests (Ginsburg, 1984). Next, the eye calibration was done. After calibration, participants performed a short practice of the road crossing task with five baseline images (no e-HMI). The experiment was divided into three consecutive sessions. After each session, there was a 30 s break. The sessions and the images within them were given in random order. Sessions included images with all combinations of car size, distance from the crossing place, and e-HMI content options. Each session contained four baseline images. Throughout the experiment, each image variation appeared three times with slight variations of the surrounding urban crossing road environment (e.g., building facade). Following the three sessions, participants were asked to explain each sign’s meaning and rate their comprehension level. Then an open interview was conducted. Then an open interview was conducted. The experimental flow is described in Figure 4.

### Eye-Tracking and Area of Interest (AOI) Definition

Eye movements and fixations data were collected and synced with the experimental timeline for each crossing scene through a designated software. Once the experiment ended, the software was used to determine whether the fixations were within the defined AOI and only that data (within the AOI) was summed per image. In the baseline images (no e-HMI), the entire image (car and environment) was defined as the AOI. For the rest, the area around the e-HMI was defined as the AOI. Its exact size was defined as the minimum size that can be expected around 0.5–1 degree in a high-end eye-tracker when the computer distance from the participant was 68.6 cm (Bojko, 2013). Hence, the AOI is defined as the multiplication of each side in the e-HMI (sign) frame length by 1.43 (see Figure 5).

### Data Analysis

A Wilcoxon test was performed to examine whether there was a difference in the dependent variables between the compatible responses and the incompatible ones. Next, a Generalized Linear Mixed Model (GLMM) was used to analyze the effect of the independent variables (message type, modality, e-HMI background color, car size, and distance) on the estimated error probability (incompatible responses, a binary logistic regression within the GLMM) of all responses over time, to examine learnability from trial to trial. Then, the effects of the independent variables were further examined on response time (ln transformed response time, a normal regression within the GLMM) and the number of fixations on the AOI over time both for all responses and for the compatible response [(ln transformed number +1), a normal regression within the GLMM]. Beyond the fixed effect, participants and image numbers were included as random effects to account for individual differences among participants and variation among images. Utilizing a stepwise process, only the main effects and the significant interactions were included in the final model. All three models used the same predicting effects- message type, modality, e-HMI background color, car size, and car distance. The final model included only significant effects or interaction related parameters.

## Results

### Crossing Decisions, Response Time, and Eye-Tracking Data

Overall, 75% (1401 out of 1867) of the decisions were compatible, and 25% (466) were incompatible. Wilcoxon tests revealed that the number of fixations for compatible responses was significantly smaller (Mean = 3.46, SD = 2.67) compared to incompatible ones (Mean = 3.85, SD = 3.02, *p* < 0.001). In addition, response time for the compatible responses was significantly shorter (Mean = 1.27 s, SD = 0.97) compared to incompatible responses (Mean = 1.50, SD = 1.25, *p* < 0.001). Table 1 shows that when the FAV is close and the e-HMI background is red, response times and the number of fixations were about twice as high in the incompatible responses compared to the compatible ones. Delving into the details, only 14 responses of all trials were incompatible (compared to 452 that were compatible in the same conditions) when the e-HMI background was red, and the car was close, and a single participant made 8 of them. This participant had dispersed response times (0.49–12.04 s), including two considerably longer ones (9.36 and 12.04 s) that occurred at the beginning of the experiment. Longer response time may imply that when pedestrians take a risk and decide to cross in close distance and red e-HMI, they tend to hesitate before crossing. It may suggest that they understood the risks of crossing and decided to cross despite them.

**TABLE 1:** Crossing decisions number of fixations and response time.

Measurements	Compatible crossing decision				Incompatible crossing decision			
		
	Red		Green		Red		Green	
				
	Close	Far	Close	Far	Close	Far	Close	Far
**Number of fixations**								
Mean	3.25	3.52	4.39	3.13	6.14	3.57	3.78	4.06
Median	3	3	3	3	4	3	3	3
Confidence interval	0.006	0.011	0.015	0.007	0.09	0.02	0.01	0.02
**Response Time [sec]**								
Mean	1.14	1.44	1.48	1.15	3.00	1.43	1.38	1.72
Median	0.91	1.10	1.08	0.94	1.74	1.16	1.27	1.27
Confidence interval	0.00	0.01	0.01	0.00	0.06	0.006	0.01	0.01
								

### Images That Received High Incompatible Responses

**FIGURE 6:**
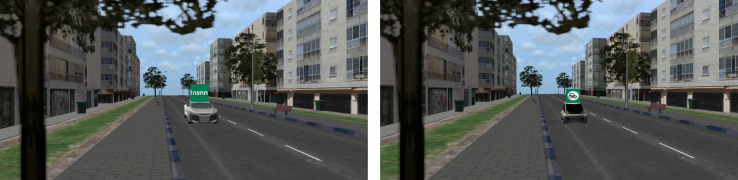
Sample images with high error rates (the right image received 50% error rates and the left 74%). The commonality amongst them was the green background e-HMI and close distance (for both symbol and text modality).

Twenty images out of the 96 images yielded an error rate of 45% or higher per image. All of these images had a green background. The FAV distance was close in eighteen of them, which implies that according to the FAV’s e-HMI, pedestrians could have crossed, but they decided not to (sample images are shown in Figure 6). One specific symbol message (the car slowing status symbol, see Figure 6) on the right) received the highest error rate. This symbol was also the lowest-ranked in the comprehensive subjective ratings (average score of 3.6 out of 10).

### Estimated Error Probability in General and Over Time

#### Distance and Color

**FIGURE 7:**
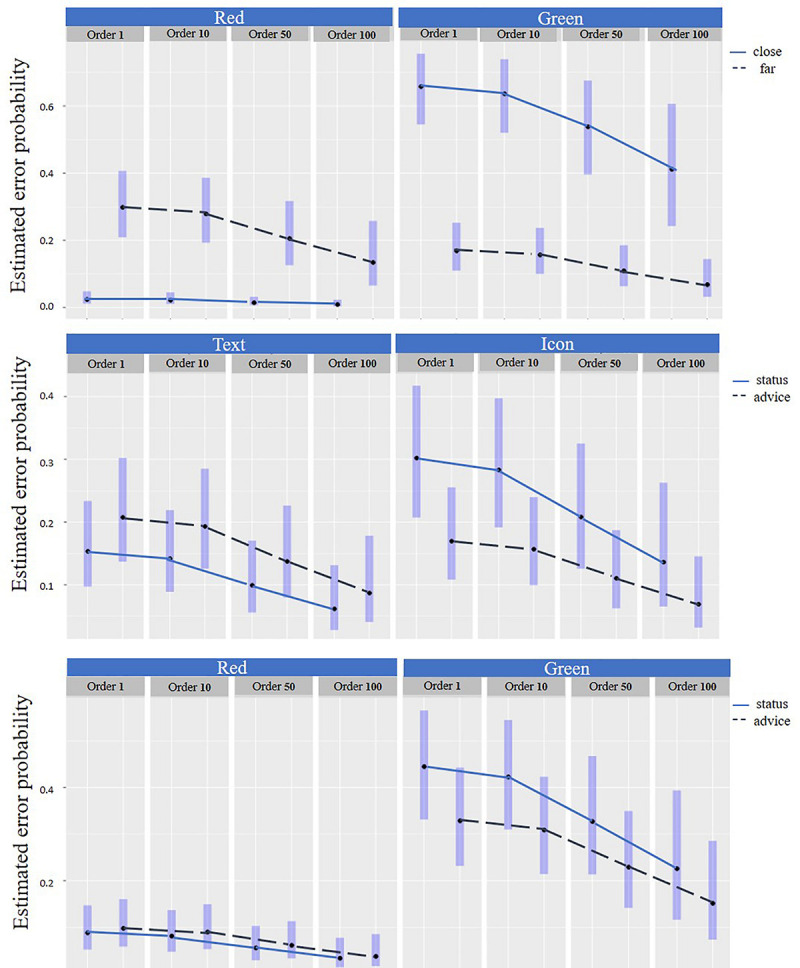
Estimated error probability. Top: By distance and color over time. Middle: By message type and modality over time and Bottom: By message type and color over time. Note: in the graphs, for visualization only, the estimated error probability was sampled in 4 chronologic locations (order) – after the first impression (image #1), at the beginning (after image #10), middle (following image #50) and at the end of the experiment (image #96), and the estimated error probability average is displayed for each sample.

A significant interaction was found between the color the distance in the estimated error probability [χ^2^ (df = 1) = 185.3, *p* < 0.001]; see Table 2 and Figure 7 Top. In the close distance, there was a significant difference in the estimated error probability between the e-HMI colors, compared to the far distance. *Post hoc* (Tukey’s-HSD) analyses revealed that in the close distance, the red background e-HMI had a significantly higher probability of compatible responses compared to the green background e-HMI (*z* = −13.63, *p* < 0.001). An opposite trend was found in the far distance; the estimated error probability in the green e-HMI was much lower from the red in the far one (*z* = 4.1, *p* < 0.001). Also, overall, results revealed a strong interaction between the fixed image order (each image had a random chronological location in each trial) and the estimated error probability. It was found that the estimated error probability reduced over time for each color – distance combination [χ^2^ (df = 1) = 11.07, *p* < 0.001]; that is, there was a learning effect over time. See Figure 7 top.

**TABLE 2:** The effect of e-HMI related factors (message type, modality, color) and crossing context factors (car size, distance, and order) on the Estimated error probability (GLMM).

	Estimated error probability	
	
Factors	χ^2^ (df = 1)	*p*-value
Order	11.07	0.00***
Message type	3.74	0.053
Modality	8.56	0.003**
Color	13.11	0.00***
Car size	0.27	0.60
Distance	32.31	0.00***
Message type * Modality	17.59	0.00***
Car size * Distance	17.27	0.00***
Message type * Color	4.9	0.03*
Modality * Color	9.52	0.002**
Car size * Color	7.88	0.005**
Distance * Color	185.3	0.00***

The χ^2^ ratio is a measure of the overall significance of the model. *p < 0.05, **p < 0.01, ***p < 0.001.

#### Modality, Message Type, and Color

It was found that there was an interaction between the message type and the color [χ^2^ (df = 1) = 4.90, *p* < 0.05], see Table 2. *Post hoc* (Tukey’s-HSD) analyses revealed that there was no significant difference in the estimated error probability in the red background e-HMI for the different message types (*z* = −0.51, *p* = 0.6) (Figure 7 Bottom). This finding means that when there was a red background e-HMI, pedestrians tended to decide not to cross in both messages type. However, in the green background e-HMI, pedestrians had higher errors when they received status messages compared to advice messages (*z* = 2.9, *p* < 0.05) (Figure 7 Bottom). Also, there was an interaction between the message type and the modality [χ^2^ (df = 1) = 17.59, *p* < 0.001] (Figure 7 Middle). In the status message, the estimated error probability for text messages was lower than for symbol messages (*z* = −4.5, *p* < 0.001). A learning effect over time that is being reflected by the reduction of the estimated error probability seems to have a similar pattern for each message type-modality combination (Figure 7 Middle) and each message type-color combination (Figure 7 Bottom).

### Response Time in General and Over Time

#### Distance and Color

**FIGURE 8:**
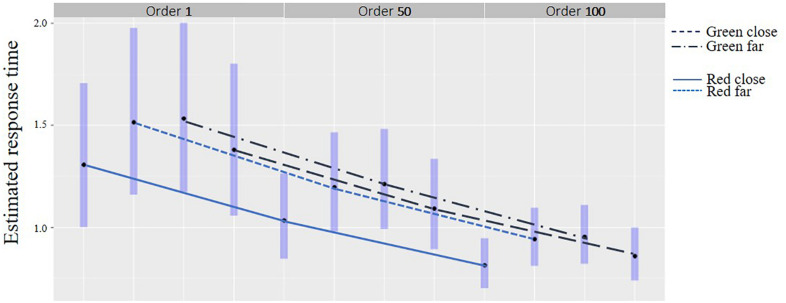
Response time for compatible responses by distance and color over time.

There was an interaction between the distance and color for response time of the compatible responses [*F*(1,1389) = 34.0, *p* < 0.001] see Table 3. *Post hoc* (Tukey’s-HSD) analyses revealed that in the close distance, response times for compatible responses were shorter for the red background e-HMI color (Mean = 1.14 s, SD = 0.71) compared to the green (Mean = 1.48 s, SD = 1.18, *p* < 0.001), as shown Figure 8. In the far distance, response time was shorter when the e-HMI background color was green (Mean = 1.15 s, SD = 0.69) compared to red (Mean = 1.44, SD = 1.29, *p* < 0.05). Overall, response time was shorter over time for each combination of distance-e-HMI background color, for all responses [*F*(1,1389) = 38.2, *p* < 0.01] and for compatible responses [*F*(1,1389) = 37.01, *p* < 0.01], as seen in Table 3 and Figure 8. Thus, there was a learning effect over time. Moreover, the learning effect shown through the reduction of response time seems to have a similar pattern for all four distance-color combinations.

**TABLE 3:** The effect of color and crossing context factors (car size, distance, and order) on Response time (ln transformed) and number of fixations for all responses and the compatible responses (GLMM).

	Response time		Number of fixations	
		
	All responses	Compatible responses	All responses	Compatible responses
**Factors**	*F*(1,1855)	*F*(1,1389)	*F*(1,1855)	*F*(1,1389)
Order	38.20**	37.01***	12.00*	11.18*
Car size	–	–	6.55*	6.40*
Distance	1.12	0.17	16.60***	15.73***
Color	2.74	4.32*	5.58*	5.54*
Distance * Color	39.06***	34.0***	20.62***	20.42***

The F ratio is a measure of the overall significance of the model.*p < 0.05, **p < 0.01, ***p < 0.001.

### Number of Fixations in General and Overtime

#### Distance and Color

**FIGURE 9:**
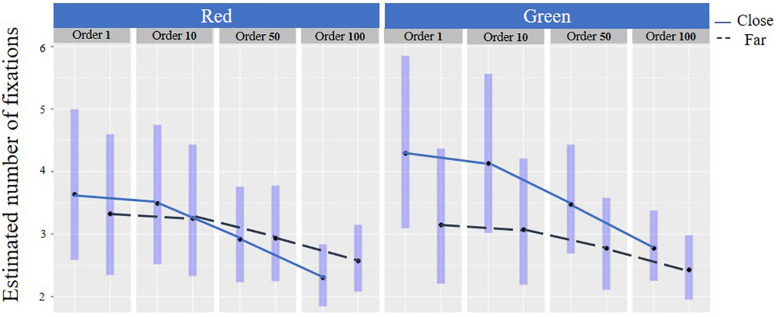
Number of fixations for compatible responses: By distance and color over time.

In general, there was an interaction between the color, distance, and the number of fixations [*F*(1,1389) = 20.42, *p* < 0.001] for the compatible responses (Table 3 and Figure 9). *Post hoc* analysis revealed that in the close distance, there were less fixations on the red e-HMI background (Mean = 3.25 SD = 2.27) compared to the green one (Mean = 4.39, SD = 3.43, *p* < 0.001). Findings reveal that for both colors, the number of fixations was reduced over time [*F*(1,1389) = 11.18, *p* < 0.05], which indicates upon learnability. However, in the close distance, the number of fixations was reduced more notably compared to the far distance. In other words, the learnability overtime was more significant in the close distance compared to the far distance (Figure 9).

#### Rate of Fixations per Millisecond

One can rightfully argue that the number of fixations will increase if response time increases, which is why it is also necessary to look at the rate of fixations. This is a similar analysis to the one in 3.5.1 of the number of fixations but now with response time as a covariate, leading to an examination of the rate of fixations per millisecond. If the response time as a covariate in the model is statistically significant, it implies that the fixation rate changes over time. Depending on the estimated mean of this covariate, one can identify the rate of change in the number of fixations over time. If the rate estimate is less than one, it means that as response time increases, the increase in the number of fixations decreases (indicating that fixations are becoming longer). Oppositely, if the rate estimate is larger than one, it indicates that the number of fixations increases as the time progresses (indicating a more erratic movement of the eyes).

The final statistical model yielded the following significant effects: Learnability over time remained significant [*F*(1,1378) = 7.24, *p* < 0.007], main effects for car size [*F*(1,1374) = 4.54, *p* < 0.033] distance [*F*(1,1375) = 24.09, *p* < 0.001], and an interaction for color and modality [*F*(1,1375) = 8.98, *p* < 0.003]. Most importantly for this analysis, response time as a covariate was statistically significant [*F*(1,1392) = 1272.03, *p* < 0.00001], indicating that indeed the rate of fixations changes over time. The estimated rate was 0.663 (SE = 0.018) thus, less than 1, indicating that as response time increases, the number of fixations increases too, but at a lower rate. Hence, most likely fixations are becoming longer in time.

## Discussion

This study aimed to explore which parameters affect pedestrians’ understanding of the FAV’s intentions as expressed in crossing decisions of participants on fixed crossing scenes, as well as the change of crossing decisions over time (learnability). Results revealed that pedestrians fixated on the FAV’s e-HMI, in line with previous research (Dey et al., 2019; Eisma et al., 2020). But, unlike what has been suggested in a previous study (Holländer and Butz, 2019), pedestrians do not always base their decision on the e-HMI proposition as demonstrated through the e-HMI background color, or message type - instruction or status. It was found that in 25% of the time, pedestrians made crossing decisions that were incompatible with what the e-HMI proposed. From observing the images that got the most incompatible responses, one can attain that in those, pedestrians made their decisions based on the FAV distance from the crossing place and decided not to cross when the FAV was close. Yet, the e-HMI background was green and proposed to cross. Also, it was found that when the e-HMI background was red and the distance was far, pedestrians sometimes decided to take the risk and cross (Figure 7 Top). This finding is in line with previous research that explored the effect of distance on pedestrians’ crossing decision (Clamann et al., 2017).

Nevertheless, when pedestrians made the compatible crossing decision when the FAV was close and the e-HMI was green, they lingered and did not decide to cross immediately. These findings were pronounced by longer response times and a higher number of fixations compared to green background e-HMI in the far distance (Figure 8 and Table 3). Also, when the FAV was far, and a red background e-HMI appeared, pedestrians also hesitated and took some time to decide (Table 3 and Figure 9). These results imply that, most likely, pedestrians base their decisions on a combination of distance and the e-HMI proposition. These findings can be explained by color conventions and distance. When the color convention fits the pedestrian’s expectations and risk due to the car’s distance, fewer fixations were needed. However, in cases where the e-HMI color convention conflicted with pedestrians’ expectations, it was necessary to further gaze on the e-HMI to understand the message and take more time to decide (Figure 8 and Table 3). These findings confirm *h1* that the e-HMI can help make the compatible decision when there are conflicts but not always, as shown in the 25% of the incompatible responses.

### Learnability

Overall, there was a learning effect over time for the various fixed effects. This was reflected in the reduction in error probability over time (Figure 7 and Table 2), as well as in the shortening of response times and the reduction in the number of fixations over time in all conditions (Figures 8, 9 and Table 3). The learning curve seems to have a similar pattern for all combinations of conditions crossing conditions (e.g., distance-color combinations). These findings are aligned with previous research findings regarding the learning effect over time (Faas et al., 2020) and strengthen them. Thus we can confirm *h3* that there is a learning effect regardless of the crossing context and e-HMI display content.

### Message Type, Modality, and Color

Results revealed that the green background e-HMI for advice message tended to be more intuitive since it had a lower estimated error probability than the green background for e-HMI status message (see Table 2). Also, in the advice message, there was no difference between the two modalities (see Figure 7 Bottom). These results confirm *h2* and can be explained by the fact that pedestrians today are more familiar with advice messages, in both modalities, and not familiar with status messages in general and with regard to FAVs. Further, it was easier to express a status message through text than through symbols, but this may change in the future when symbols become more standardized and common.

This study sheds more light on the contradicting findings of previous studies and emphasizes that pedestrians are not yet in a stage where they trust FAV e-HMI entirely in contrast to some findings (Holländer and Butz, 2019). However, they do not ignore it (in contrast to Clamann et al., 2017; Mahadevan et al., 2018). This study revealed, from analyzing the number of fixations and response time, that pedestrians tend to decide for themselves whether to cross the road based on a combination of the FAV distance from the crossing place and the e-HMI background color and instructions.

Our study highlights the importance of the e-HMI and how it may affect pedestrians’ decision to cross. However, several limitations must be noted. A major limitation is in the crossing conditions of only one FAV and one pedestrian at a specific time, unlike the real world. Another limitation refers to the form of presentation, that is, the fixed scenes. Although this form allows us to examine pedestrian behavior parameters (such as understanding) more deeply, it ignores other parameters that are associated with the dynamicity of the road crossing task. Lastly, the study population included a convenience sample of students. Future studies should examine our findings in dynamic scenarios and with more complex and varied crossing conditions such as multiple FAVs on the road, different car types, etc. Further, pedestrians’ decisions may be influenced by the presence of other pedestrians, which we did not examine. Last but not least, as shown in pedestrian studies (e.g., Tapiro et al., 2016, 2020), findings must be further evaluated across cultures and with regard to children and older adults. Finally, while this study addressed learnability, we still do not know enough about how and if the eHMI proposition will lead pedestrians to behave in compliance with its recommendation even in conflict situations, to establish this, we need to examine the learnability curve further using varied learnability inflators, such as system errors, misses and false alarms, varying trust level, etc.

## Conclusion

Over time, learning was apparent in response times and gaze for crossing context and e-HMI characteristics combinations for the compatible and all responses. Therefore, it is essential to provide e-HMI designs that will minimize error probability and provide fast response times, and need for only a minimal number of fixations on the e-HMI from the first phase. The existence of learning is encouraging, as it implies that crossing performance can be improved over time. Further, color conventions play a significant role in pedestrians crossing decisions today, and they will probably influence decisions in the FAV world as well, at least in the upcoming decade. This finding is important and emphasizes that it is essential to adhere to existing color convention, in line with Bazilinskyy et al. (2020) and not use neutral colors for all FAV messages, as some researchers have suggested (Dey et al., 2020). Yet, pedestrians also considered the FAV’s distance from the crossing place when deciding to cross, especially in conflict situations. This skill of estimating the risk of the crossing from the distance of the vehicle is necessary for today’s pedestrians. Still, it may diminish in the future FAV world if and when pedestrians will over trust the e-HMI and base their decisions solely on its recommendations and status. This research used fixed scenes, which allows examining in-depth, how pedestrians related to the crossing scene over time in the FAV world and how the e-HMI influenced their decision. However, to extend these findings, it is necessary to conduct further studies with dynamic various crossing complexities, examine further learnability inflators, and include diverse, multicultural populations, such as the elderly and children.

## Data Availability Statement

The original contributions presented in the study are included in the article/Supplementary Material, further inquiries can be directed to the corresponding author.

## Ethics Statement

The studies involving human participants were reviewed and approved by the Department Internal Review Board, Department of Industrial Engineering and Management, Ben-Gurion University of the Negev. The participants provided their written informed consent to participate in this study. Written informed consent was obtained from the individual(s) for the publication of any potentially identifiable images or data included in this article.

## Author Contributions

All authors listed have made a substantial, direct and intellectual contribution to the work, and approved it for publication.

## Conflict of Interest

The authors declare that the research was conducted in the absence of any commercial or financial relationships that could be construed as a potential conflict of interest.

## Footnotes

1 https://www.mak.com/
