# Supplementary figures and images for: Pedestrians’ Understanding of a Fully Autonomous Vehicle’s Intent to Stop: A Learning Effect Over Time
Source: Front Psychol. 2020 Dec 3;11:585280. doi: 10.3389/fpsyg.2020.585280 (PMC7744601; doi:10.3389/fpsyg.2020.585280)

**Appendix 1 - Images content (1-108)**


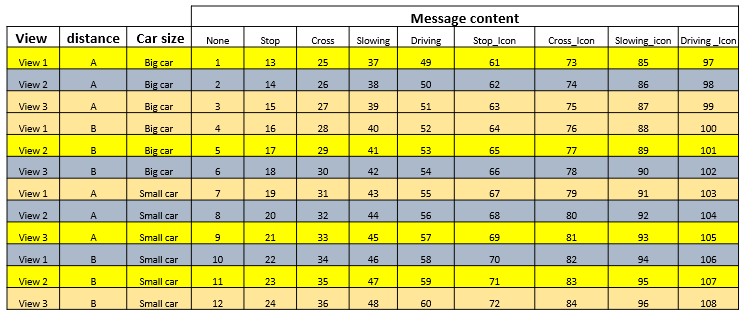

Supplement: Supplementary file 1 [file Table_1.DOCX]
